# EfficientNet-based machine learning architecture for sleep apnea identification in clinical single-lead ECG signal data sets

**DOI:** 10.1186/s12938-024-01252-w

**Published:** 2024-06-20

**Authors:** Meng-Hsuan Liu, Shang-Yu Chien, Ya-Lun Wu, Ting-Hsuan Sun, Chun-Sen Huang, Kai-Cheng Hsu, Liang-Wen Hang

**Affiliations:** 1 Artificial Intelligence Center, China Medical University Hospital, No. 2, Yude Rd, North Dist, Taichung, Taiwan; 2 Sleep Medicine Center, Department of Pulmonary and Critical Care Medicine, China Medical University Hospital, No. 2, Yude Rd., North Dist, Taichung, Taiwan; 3 School of Medicine, China Medical University, Taichung, Taiwan; 4 Neuroscience and Brain Disease Center, China Medical University, Taichung, Taiwan; 5 Department of Neurology, China Medical University Hospital, Taichung, Taiwan; 6 Department of Respiratory Therapy, College of Health Care, China, Medical University Hospital, Taichung, Taiwan

**Keywords:** Sleep apnea, Single-lead electrocardiograph signals, Short-time Fourier transform, Deep learning, Machine learning

## Abstract

**Objective:**

Our objective was to create a machine learning architecture capable of identifying obstructive sleep apnea (OSA) patterns in single-lead electrocardiography (ECG) signals, exhibiting exceptional performance when utilized in clinical data sets.

**Methods:**

We conducted our research using a data set consisting of 1656 patients, representing a diverse demographic, from the sleep center of China Medical University Hospital. To detect apnea ECG segments and extract apnea features, we utilized the EfficientNet and some of its layers, respectively. Furthermore, we compared various training and data preprocessing techniques to enhance the model’s prediction, such as setting class and sample weights or employing overlapping and regular slicing. Finally, we tested our approach against other literature on the Apnea-ECG database.

**Results:**

Our research found that the EfficientNet model achieved the best apnea segment detection using overlapping slicing and sample-weight settings, with an AUC of 0.917 and an accuracy of 0.855. For patient screening with AHI > 30, we combined the trained model with XGBoost, leading to an AUC of 0.975 and an accuracy of 0.928. Additional tests using PhysioNet data showed that our model is comparable in performance to existing models regarding its ability to screen OSA levels.

**Conclusions:**

Our suggested architecture, coupled with training and preprocessing techniques, showed admirable performance with a diverse demographic dataset, bringing us closer to practical implementation in OSA diagnosis.

*Trial registration* The data for this study were collected retrospectively from the China Medical University Hospital in Taiwan with approval from the institutional review board CMUH109-REC3-018.

**Supplementary Information:**

The online version contains supplementary material available at 10.1186/s12938-024-01252-w.

## Introduction

### Background

Poor quality of sleep is well known to not only cause mood disorders, but also lead to a higher incidence of traffic accidents. Obstructive sleep apnea (OSA), a type of sleep disorder, adversely affects health, and an increasing body of evidence has demonstrated that OSA has a high correlation with hypertension, coronary artery disease, heart failure, arrhythmia, and stroke. The most common reason for OSA is an oxygen supply shortage caused by pharyngeal collapse during sleep, which increases the burden on the cardiovascular system and causes problems related to the system [1–3]. To enable diagnosis of OSA, polysomnography (PSG) was developed, with nasal airflow and thoracic abdominal effort used as the main measurements, through which the average number of apnea and hypopnea events per hour of sleep (the apnea–hypopnea index [AHI]) can be obtained. PSG methods include electrocardiography (ECG), peripheral oxygen saturation (SPO2) monitoring, electroencephalography (EEG), and electrooculography (EOG) [4, 5]. However, traditional methods for diagnosing OSA can only be employed under specific conditions. In addition to equipment for at least 12 types of measurements, traditional methods require a suitable sleeping environment and qualified personnel to manage the measurement processes, which raises the threshold for OSA diagnosis. To solve these problems, scholars have sought methods for identifying apnea patterns through single-signal measurements, such as SPO_2_ [6–9] monitoring, heart rate monitoring [10–12], and ECG. Among these, ECG has gained the most attention because ECG data are informative and easily obtained. At the beginning, researchers often use pure signal preprocessing and feature engineering-based method to identify features of OSA events through standard signals. These rules are based on a deep understanding of the underlying principles and physics of the problem, and are often tailored to specific applications. Fourier transforms and wavelet transforms have commonly been employed for signal preprocessing [13–17]. Some statistical methods, such as mean absolute deviation and entropy, are subsequently applied to prepare the features for computer classification. In recent years, the rise of artificial intelligence has provided us more options to detect the apnea signal.

### Relevant studies

Several studies have demonstrated the effectiveness of deep learning algorithms in enhancing apnea prediction when applied to engineered features. For example, Wang et al. [18] developed a deep learning model to identify patterns in R–R interval (RRI) signals and R-wave amplitude (RA) derived from the raw ECG signal using engineering-based algorithms. Shen et al.[19] built a model that combined a multiscale dilation attention 1-D convolutional neural network (MSDA-1DCNN) and a weighted-loss time-dependent (WLTD) classification model. This approach effectively addressed the issues arising from nonstationary sympathetic nerve signals and complex heart rate variability (HRV) characteristics. Similarly, Qin et al. [20] used the extracted RRI signal in a dual deep-learning model, using the ADASYN sampling method to handle data imbalance. Yang et al. [21] opted to include multiple signal features, such as RA, RRI, and Q-amplitude (QA), in the AI inference.

In addition to supervised deep learning methods for analyzing engineering-based features, some studies have used unsupervised methods to obtain effective feature representations with unlabeled data. For instance, Li et al. [22] used a sparse auto-encoder to learn features from the unlabeled RR interval and support vector machine (SVM) and artificial neural network (ANN) classifiers to categorize the extracted features. Feng et al. [23] used a similar approach, employing a frequency-based stacked sparse auto-encoder to extract features from RR interval and a time-dependent cost-sensitive model to identify apnea events.

Some studies have directly used the single-lead ECG signal as the input for the deep learning model. Dey et al. [24] and Chang et al. [25] proposed a deep learning framework that included a convolutional neural network (CNN), eliminating the need for separate feature extraction and classification algorithms. Mashrur et al. [26] used the hybrid scalogram as the input to their lightweight CNN model. Given that long short-term memory (LSTM) can learn temporal information from sequences of inputs, some studies [27–30] have combined CNN and LSTM models to detect apnea. Most literature suggests that the combination of these two models is superior to using a single model alone. For example, Sheta et al. [29] conducted comprehensive research on machine learning and deep learning-based methods applied to apnea detection, with experimental results favoring the CNN-LSTM model.

Adopting raw ECG signals and extracted features simultaneously into the model has also proven effective in some literature. For instance, Hu et al. [31]constructed a transformer-based model with multichannel inputs, where the attention mechanism helps to find the relative important feature. Furthermore, they also employed a semi-supervised fine-tuning method in subsequent publications to facilitate low-cost implementation of personalized models [32, 33].

In our study, we aimed to develop an apnea-detection workflow for processing extensive single-lead ECG data from the China Medical University Hospital (CMUH) sleep center. To enhance our model’s performance and save computation consumption, we only utilized short-time Fourier transform (STFT) to gain features of the signal in the frequency and time domain. In terms of the model, we employed machine learning (ML) and EfficientNet-based models for classification and feature extraction, respectively. The simple preprocessing method and hybrid architecture perform well in our extensive data set.

The rest of the paper is structured as follows: Sects. “Results” and “Discussion” test the system’s performance with different data and compare its performance with similar systems. Sect. "Conclusions" concludes the paper and offers suggestions for future models. Finally, Sect. "Materials and methods" introduces our training data, presents a simple method for data preprocessing, and outlines the roles of ML and DL in the proposed system.

## Results

The outcomes of this study is detailed in this section. The functions of the model (apnea event detection and OSA level screening) are tested using the ECG records of the 276 patients with OSA, which were obtained from the CMUH sleep center. The effectiveness of the model is evaluated using ACC, SP, SE, and AUC.

### Per-segment classification of apnea and nonapnea

Initially, we examine the performance of the models trained under different conditions. To observe their functioning on patients with varying levels of severity, we also divided the test data into groups according to the physician-diagnosed severity of OSA patients. The performance of the model is presented in Tables 1, 2, 3. When we trained the model using the class weight with non-overlapping slicing data (Table 1), the average ACC, AUC, and SP reached 0.874, 0.900, and 0.963, respectively, although the SE achieved only 0.612. For the low-level OSA groups, the SE was low. For example, the SE in the AHI < 5 group was only 0.198. This indicates that the ratio of apnea to nonapnea segments differed considerably between OSA levels, which created problems when we trained the model. Therefore, to solve the problem, we used the sample weight rather than the class weight.

**Table 1:** Results of class weight and non-overlapping slicing training

Groups	Number of segments	ACC	SE	SP	AUC
ALL	(*N* = 11,3525)	0.874	0.612	0.963	0.900
AHI < 5	(*N* = 45,338)	0.948	0.198	0.975	0.779
AHI 5–15	(*N* = 30,381)	0.842	0.304	0.951	0.775
AHI 15–30	(*N* = 15,285)	0.793	0.502	0.959	0.858
AHI > 30	(*N* = 22,521)	0.823	0.785	0.927	0.931

**Table 2:** Results of sample weight and non-overlapping slicing training

Groups	Number of segments	ACC	SE	SP	AUC
ALL	(*N* = 11,3525)	0.854	0.727	0.897	0.900
AHI < 5	(*N* = 45,338)	0.897	0.464	0.913	0.831
AHI 5–15	(*N*= 30,381)	0.825	0.523	0.886	0.816
AHI 15–30	(*N* = 15,285)	0.815	0.696	0.883	0.874
AHI > 30	(*N* = 22,521)	0.834	0.827	0.853	0.914

**Table 3:** Results of sample weight and overlapping-slicing training

Groups	Number of segments	ACC	SE	SP	AUC
ALL	(*N* = 22,6613)	0.855	0.787	0.878	0.917
AHI < 5	(*N* = 90,513)	0.882	0.521	0.894	0.843
AHI 5–15	(*N* = 60,646)	0.821	0.570	0.871	0.828
AHI 15–30	(*N* = 30,502)	0.825	0.760	0.861	0.896
AHI > 30	(*N* = 44,952)	0.872	0.888	0.829	0.936

Through this, the ratio of the positive and negative (P/N) segments in each patient’s signal was taken into account to adjust the weight, which also enabled the model to learn rare apnea features present in low-level OSA signals. This significantly improved the sensitivity of the model, as indicated in Table 2, in which the sensitivity is notably improved for low OSA levels, with a gain of 26.5%.

Overlapping data slicing also enabled the model to learn effectively from the training data. In the sample-weight, non-overlapping data trial, only ACC = 0.854, AUC = 0.900, and SP = 0.897 were achieved. Overlapping slicing can increase the probability of complete apnea signals being included in segments, and it enables models to be fed a double amount of data. For the overlapping trial (Table 3), we developed a postprocessing smoothing method that normalizes the prediction score of each segment with those of the previous and subsequent sections to create a continuous record for the entire night. This prevents potential contradictions, such as one segment containing an apnea signal when the neighboring, overlapping segments do not. Overlapping data slicing yielded the most favorable performance, with ACC = 0.855, AUC = 0.917, and SP = 0.878. The results of the subsequent, more-detailed trial are displayed in Table A.1 in the Online Appendix.

### Per-record OSA level screening

Rather than using the detected apnea events divided by the total recorded time to assess the severity of OSA, as AHI has been performed in the literature [18, 22, 25, 28, 34], we used our trained DL model to extract features of patient sleep records for more accurate identification of the patients’ sleep states. The results of the different trials are displayed in Tables 4, 5. Under the same ML model setting, the results demonstrate that the model’s performance improved as the screening OSA threshold increased, indicating that signals from patients with higher levels of OSA have clearer OSA patterns. This also explains why the sensitivity of the per-segment classification improves as the severity of the groups increases. Additionally, the results revealed that the model performed best with overlapping slicing and the sample weight set to the results of per-segment classification. When we used the SVM model as a classifier, for the AHI > 15 screening, ACC = 0.899 and AUC = 0.949, and for the AHI > 30 screening, ACC = 0.917 and AUC = 0.972. When we used the XGBoost model, for the AHI > 15 screening, ACC = 0.909 and AUC = 0.961, and for the AHI > 30 screening, ACC = 0.928 and AUC = 0.975. These results demonstrate that the XGBoost model performed more favorably than the SVM model did. In our previous study, we applied a method for OSA level screening that combines the statistical results of SPO_2_ data with an SVM model [7]. However, in this study, we used a trained DL model as a feature extractor in combination with an ML model to deal with ECG data. Through this proposed model, the significance of ECG data may be comparable to that of SPO_2_ data in OSA level screening, with the results for SPO_2_ data indicating ACC = 0.880 and AUC = 0.941 for AHI > 15 screening and ACC = 0.904 and AUC = 0.958 for. AHI > 30 screening [7].

**Table 4:** OSA level screening in SVM model

Threshold	ACC	SE	SP	AUC
AHI = 5	0.746	0.661	0.880	0.886
AHI = 15	0.899	0.851	0.923	0.949
AHI = 30	0.917	0.929	0.914	0.972

**Table 5:** OSA level screening in XGBoost model

Threshold	ACC	SE	SP	AUC
AHI = 5	0.812	0.804	0.924	0.901
AHI = 15	0.909	0.862	0.934	0.961
AHI = 30	0.928	0.857	0.945	0.975

## Discussion

### Comparison with overlapping-slicing and general slicing

Through the mathematic derivation, we can get the probabilities that a window can completely cover a particular apnea signal under different slicing methods. For the general slicing, the probability is

1 $$P\left({w}_{s},x\right)=\left\{\begin{array}{c}\frac{{w}_{s}-x}{{w}_{s}}, x\le {w}_{s}\\ 0, x>{w}_{s}\end{array},\right.$$

where ${w}_{s}$ is the slice window length and the *x* is the apnea signal length. For the overlapping slicing, the probability is

2 $$P\left(w,x\right)=\left\{\begin{array}{c}1, x<\frac{1}{2}{w}_{s}\\ \frac{2\left({w}_{s}-x\right)}{{w}_{s}}, {w}_{s} \ge x >\frac{1}{2}{w}_{s}\\ 0, x>{w}_{s}\end{array}.\right.$$

With the apnea length distribution of our data set (see Fig. 2), we can estimate the posterior probability that our window could cover the whole apnea signal with the marginalization method [35]:

3 $$P\left({w}_{s}\right)={\int }_{10}^{\infty }P\left({w}_{s},x\right)D\left(x\right)dx,$$

where *D*(*x*) is the probability distribution of the apnea length. For the general slicing, the result showed that the window with a length of 60 s could only cover the 54.3% apnea event in the slicing. And for the window length = 30 s only has 19.4% to cover the complete apnea signal. To make most of the signal could be fully covered in at least one segment, we adopted the overlapping slicing in our literature. Under this method, the probabilities that at least one segment can fully cover the apnea signal are 89.2% with window length = 60 s and 38.0% with window length = 30 s.

With the above calculation, we could know that the overlapping slicing helps cover the entire apnea signal. And the test results shown in Table 2 and Table 3 also prove the setting with overlapping slicing has superior performance to the non-overlapping slicing one.

### Models applied to data from PhysioNet Apnea-ECG database

We tested the proposed model on data from the PhysioNet Apnea-ECG database, a well-established benchmark dataset commonly used for evaluating apnea-detection models [36, 37]. In order to adapt the model effectively to the features of the PhysioNet data, we employed a fine-tuning approach. Initially, we initialized the model weights with pre-trained weights obtained from the CMUH Model and utilized the Adam optimizer with an initial learning rate of 1e−5. The fine-tuning process allowed the model to better adapt to the specific features of the PhysioNet dataset. By leveraging the fine-tuning approach we adopted, we aimed to minimize potential biases or discrepancies arising from using data from different sources.

#### Per-segment classification

Although the overlapping-slicing training could not be applied to this 1 min, labeled data set, the results of the trial reveal AUC = 0.944 and ACC = 0.888 for apnea detection, which is higher than the results for the CMUH data set. This is likely due to the population of the data having a strong influence on the results. Compared with those in the CMUH data, most of the PhysioNet cases were distributed at normal and severe levels, making it much easier for the model to distinguish between normal and OSA segments. In addition, the large population of the CMUH data was challenging for the model to process because the diversity in the ECG signals can increase the likelihood of distortion in apnea detection, although the results of large populations are much closer to those of real-world applications.

#### Per-record classification

**Fig. 1:**
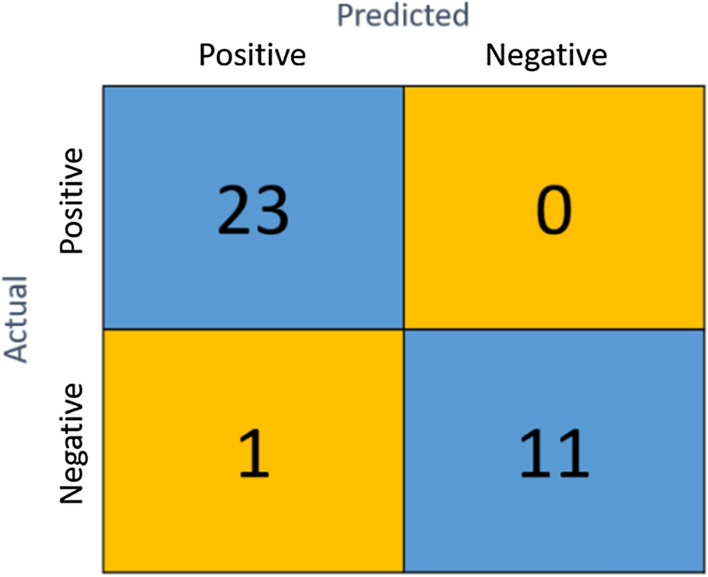
Confusion matrix of AHI > 5 OSA level in PhysioNet data using the EfficientNet + SVM classifier

The confusion matrix for the AHI > 5 screening for the PhysioNet data is displayed in Fig. 1. Only 1 FP occurred in the 35 test records, indicating ACC = 0. 971, SE = 1.000, and SP = 0.917. In adopting only linear SVM for this small data set, we achieved results comparable to those of other studies, the performances of which are summarized in Table 6.

**Table 6:** Comparison of our model with elated studies for per-record screen (threshold: AHI = 5) of PhysioNet data

Paper	Year	Input signal	Model	ACC	SEN	SPC
Li et al. [22]	2018	RRI	Sparse auto-encoder + SVM, ANN	1.000	1.000	1.000
Wang et al. [18]	2019	RRI and RA	Modified LeNet-5	0.971	1.000	0.917
Chang et al. [25]	2020	ECG signal	1-D Deep convolution neural network model	0.971	0.951	1.000
Feng et al. [23]	2020	RRI	FSSAE + TDCS	0.971	0.951	1.000
Shen et al. [19]	2021	RRI	MSDA-1DCNN + WLTD	1.000	1.000	1.000
Mashrur et al. [26]	2021	Scalogram	Scalogram-based convolutional neural network	1.000	1.000	1.000
Zarei et al. [28]	2021	ECG signal	CNN-LSTM	1.000	1.000	1.000
Qin et al. [20]	2021	RRI	ADASYN + 1DCNN-RLM + BiGRU-TDM	1.000	1.000	1.000
Yang et al. [21]	2021	RRI, RA and QA	1D-SEResGNet	1.000	1.000	1.000
Hu et al. [31]	2022	ECG signal RA, RRI, RRID	MPCA + transformer	1.000	1.000	1.000
Our study	2024	Spectrogram	EfficientNet B0 + SVM	0. 971	1.000	0.917

#### Comparison with other studies

In comparison with other studies, our approach to addressing similar objectives differs significantly in several key aspects. We applied intuitive techniques, such as short-time Fourier transform (STFT), to convert raw electrocardiogram (ECG) data into two-dimensional images, thereby enhancing training effectiveness through innovative overlapping slicing methods. Additionally, we mitigated the pronounced imbalance in positive and negative class ratios within our dataset by carefully selecting sample weights instead of relying on traditional class weight adjustments.

Furthermore, we observed that many studies do not transform ECG data into images but rather analyze raw signals and extracted features [18–23], such as R-peaks (RA, RRI, RAID, etc.), achieving satisfactory results. Regarding model architecture, while we utilized the EfficientNet framework for image classification, other studies, exemplified by Feng et al. [23], demonstrated significant achievements using different Convolutional Neural Network (CNN) models. In non-CNN deep learning architectures, pioneering research by the studies [22, 23, 31, 33] explored the use of attention-based Transformer frameworks, yielding promising results in classification tasks.

Similarly to our labeling preprocessing, Hu et al. [32] tackled an issue by varying the mapping labeling length (including surrounding signal segments while keeping the labels of the central 1-min segment). They found that increasing the mapping labeling length positively affected the model’s behavior. These outcomes suggest that the completeness of the signals indeed influences the model’s capability.

Hence, the methodological differences underscore the multifaceted nature of addressing similar research objectives, with each approach offering unique advantages and challenges. This highlights the importance of further exploration and research in diverse directions within the computational analysis of ECG data.

### AHI value evaluation

For the per-record classification, it is common to evaluate the predicted apnea segments divided by the total record time to assess the AHI value, as has been performed in other studies. However, some potential problems should be considered:

- In the real world, knowing the total sleep time is essential to calculate the AHI value. Unfortunately, if the model cannot recognize the sleep stage, it is impossible to get this value needed in the AHI formula.
- Undercount might happen when there are over two apnea events in a segment. And the overcount would occur when the same apnea signal is distributed in two neighborhood pieces.

To avoid the aforementioned worries, we used features extracted by the DL model to do the classification. It enabled us to directly diagnose the severity of OSA through patients’ physical states, as indicated by their sleep records. The method finally showed good prediction in both CMUH and the PhysioNet data set.

## Conclusions

Our study evaluated different methods of detecting OSA signals by using DL combined with ML models. The results reveal that the method with combined overlapping slicing in preprocessing and a sample-weight setting in the training process is the most suitable for large data sets, such as those from the CMUH sleep center. Overlapping slicing increased the probability that the apnea signals could be completely sliced from 54 to 89.2%, and the sample-weight setting solved the problem of the imbalance in positive/negative segments in each sleep record. Under this setting, the ACC and AUC for apnea detection per segment reached 0.855 and 0.917, respectively. For severe OSA level (AHI > 30) screening, our developed method made the ACC and AUC reach 0.928 and 0.975, respectively. To ensure that a model achieves similarity to real-world application, the model was trained with a data set of a large population and range. Our study identified problems that may arise from use of such data sets and proposed viable solutions to solve them. Because an increasing amount of data are being systematically collected with portable ECG devices, we believe that the results of this study may be applicable in the development of ECG OSA signal detection. However, application of sleep ECG data need not be limited to apnea detection. In the future, we plan to combine ECG data with PSG records of sleep stages, which are also strongly related with sleep quality, to develop a more complete model for analyzing sleep states.

## Materials and methods

### Overview

Our study methodology included the following 5 steps: (1) ECG data collection from the CMUH sleep center; (2) data preprocessing using STFT and slicing; (3) model development; (4) model training; and (5) performance evaluation.

### Data collection

The data used in our study were obtained from the sleep center of CMUH, and our study obtained institutional review board approval in 2020. After the data of 4 individuals who did not experience respiratory events, sleep arousal, decreases in SPO_2_, and periodic leg movement were excluded, data from the overnight ECG signal records of 1656 patients were included for model training and testing. Normal and apnea event periods were delineated by the technician of the center by using PSG data, which is the gold standard for diagnosing OSA. The demographics of the patient population of the collected data were of broad range, which indicates that the model was potentially trained with most types of regular cases. For example, the ages of the patients ranged from 2 to 90 years, and their body mass indexes (BMIs) ranged from 10 to 55. The demographics of the included population are listed in Table 7.

**Table 7:** Demographics of the entire, training, and test groups

Measures	Total group (*N* = 1656)				Training group (*N* = 1380)				Testing group (N = 276)			
	Mean	SD	Min	Max	Mean	SD	Min	Max	Mean	SD	Min	Max
Age (years)	38.36	18.35	2	90	38.25	18.53	2	90	38.92	17.4	2	76
BMI (kg/m_2_)	26.90	6.59	9.92	55.35	26.89	6.61	9.92	53.04	26.95	6.53	13.29	55.35
AHI (per h)	17.98	22.82	0.00	131.95	18.02	22.48	0.00	114.92	17.81	24.45	0.00	131.95
AI (per h)	10.90	19.25	0.00	122.99	10.75	18.6	0.00	112.31	11.65	22.21	0.00	122.99
HI (per h)	7.08	8.76	0.00	58.75	7.27	9.08	0.00	58.75	6.16	6.92	0.00	34.68
TST (h)	6.88	0.56	4.08	10.11	6.88	0.56	4.08	10.11	6.91	0.58	6.98	9.97
Sex (M:F:U)	1168:472:16				987:380:13				181:92:3			

*M:F:U* male:female:unknown

### Data preprocessing

In order to make the input data more informative and suitable for our proposed model, we do the data preprocessing as follows.

#### Short-time Fourier transform (STFT)

STFT is commonly used in signal processing as a method that identifies changes in frequency over time. The processed formula is shown as follows:

4 $${\text{X}}\left( {\text{t,f}} \right){ = }\mathop \smallint \limits_{{{ - }\infty }}^{\infty } w_{H} \left( {{\text{t - }}\tau } \right) \times \left( \tau \right){\text{e}}^{{{\text{ - i2}}\pi {\text{f}}\tau }} {\text{d}}\tau ,$$

where ${w}_{H}$ represents the window function in the STFT, which is the Hamming window [38]. *X* is the transformed signal, *x* represents the original ECG signal, and f and t are the frequency and the time, respectively. Once the signal has been transformed, the resulting output demonstrates how frequency varies over time, and can potentially reveal distinct characteristics of the event of interest, thus aiding in model recognition. To provide our model with more informative features, we transformed the overnight ECG record of each patient from a 1-dimensional signal into a 2-dimensional spectrogram. Here, we set the window length = 0.5 s by considering the spectrogram’s frequency and time resolution balance. The window length should be as large as possible to provide enough frequency information while still performing with the instant wave change in the signal. On the other hand, the overlap is chosen as 0.25 s to simultaneously shorten the preprocessing time and make the spectrogram smooth.

#### Slicing methods

**Fig. 2:**
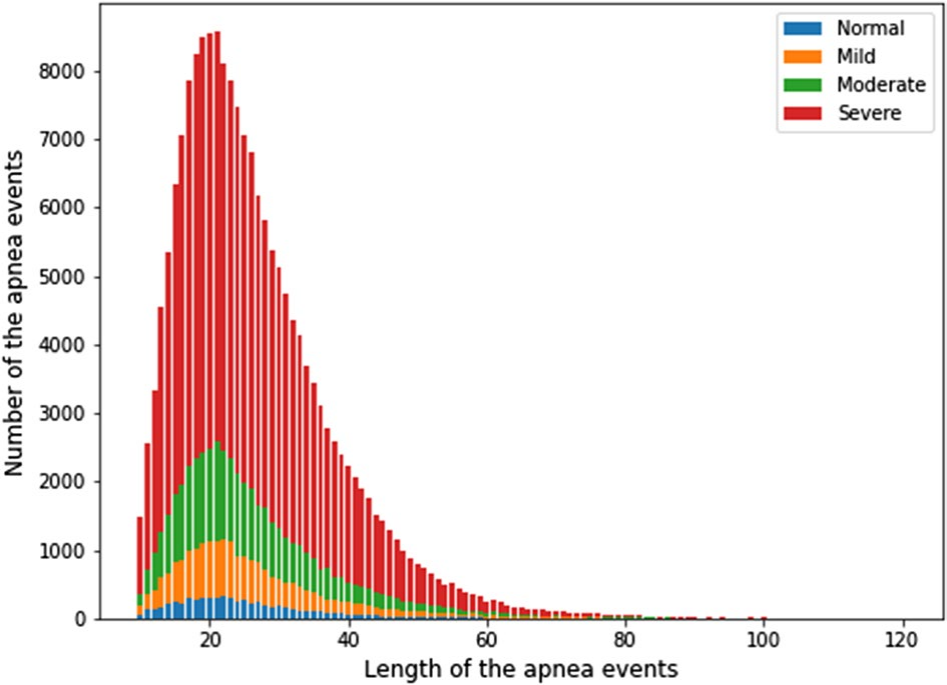
The distribution of the apnea events’ length in entire data and different severity group of the patient

The sleep data were then sliced into segments to input the model. The sliced length should be multiples of 30 s, which is restricted to clinical usage in the CMUH sleep center. Considering the fixed input pixel of the DL model, it is practical to set the window length under 1 min to avoid more information loss from a higher compression ratio. We also hope the model can learn the complete apnea signal patterns in sliced segments. As a result, most of the pieces should cover the apnea event. By statistics of our data (Fig. 2), it shows that apnea length < 30 s accounts for 70.6% of our data, but the apnea length < 60 s accounts for 98.4%. Finally, we chose the 60 s as our window length.

Furthermore, it is still possible for the slice to accidentally cut the apnea signal into pieces. Here, we adopted the overlapping slicing, which shifts the slice window to half of the segment’s length for every step to increase the chance of covering the complete apnea signal and double the training data set. The preprocessing with non-overlapping slicing is also tried, and its outcomes are also mentioned in the results for comparison.

#### Data labeling

Since our study has two tasks: per-segment classification and per-record classification, we prepare our input data and labels in different forms. For the per-segment classification, we prepare the sliced 1-min segments with the labels if the segments contain apnea events. For the per-record classification, we take a set of sliced segments of a certain patient’s whole night sleep record as the input data with the label if the patient’s OSA level is larger than a certain degree. All the preprocessing data were divided into six parts equally according to the patients’ number, with 5 for training (1380 people) and validation tasks and 1 for testing (276 people).

### Models

**Fig. 3:**
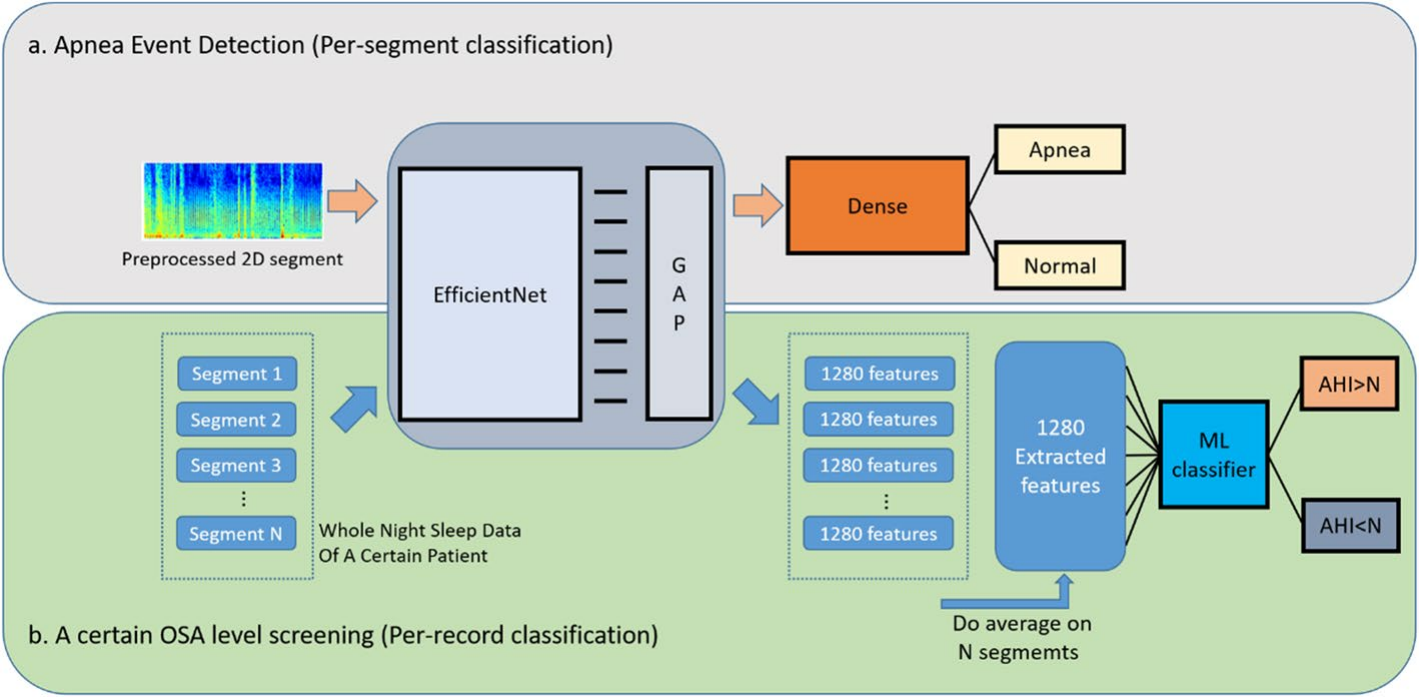
Architecture of the proposed system: **a** flowchart of apnea event detection and **b** OSA level screening (GAP = global average pooling)

We developed the architecture of our system with Keras, an open-source code that provides modules for artificial neural networks [39]. Our method, which contains the DL and ML, performs apnea event detection in the per-segment classification and OSA level screening in per-record classification. Under the architecture (Fig. 3), the DL model is not only a classifier in apnea detection. It also extracts features from the segments in the OSA level screening task. The ML model then simply uses extracted features to classify the OSA severity. The related details are as follows.

#### Deep learning model background

The EfficientNet is truly the backbone of our model structure. It is a kind of CNN-based model introduced by the Google AI team in 2019 [40]. The model comprises several functional layers which are the same as the standard CNN, such as pooling, convolutional, and dense layers [41]. However, their construction should follow the rules of “Compound Model Scaling”. It systematically increases these modules to ensure deep and wide balancing, which can enable scaling up the accuracy without wasting computing resources. Here, we chose the EfficientNET-B0 by comparing it with another testing B7 structure (see Table. A2 in Online Appendix), which shows almost the same performance but the lowest time consumption due to the convolution design minimizing parameters and increasing its processing speed.

#### Machine learning model background

Two ML models were used as a classifier in the task: the support vector machine (SVM) and XGBoost [42, 43]. XGBoost employs ensemble learning, which, due to its tree algorithm, can easily adapt data. XGBoost is often used in competitions because of its high speed and data adaptation in dealing with large samples. By comparison, the SVM model is a traditional ML model that uses a generic kernel to project data. The SVM is most effective when the number of dimensions is lower than the number of samples.

#### Architecture

The whole architecture can be easily separated as two parts: per-segment classification and per-record classification (see Fig. 3). For per-segment classification, the sliced 1 min spectrogram would be the input of the EfficientNet. And the inference result is the probability of whether the segment included an apnea event. For the per-record classification, we take the part of EfficientNet without its dense layers as the feature extractor. All 1-min ECG spectrograms of a patient’s whole night sleep record would then be transformed as the set of N*1280 features through it, where N is the number of the segments input. Next, we do an average on N*1280 features and get the 1280 mean features as the input to the ML model. Finally, the ML inference result enabled us to identify patients’ OSA levels.

### Model training

Firstly, we trained the EfficientNet, which is mainly for the per-segment classification, with the spectrogram data. The model, which has been pre-trained with the noisy student method [44], is the starting point in our training task. In the training process, we tuned the whole parameters of the architecture, enabling the model to distinguish if the segment includes apnea or not. On the other hand, weight adjustment methods are also adopted here to solve the individual and overall imbalances in the positive/negative ratio, which brought the prediction bias of the model. The results of the sample weight and class weight adjusted in training were shown in the literature. The classic weight value of each segment was decided according to the ratio of the amounts of positive and negative pieces for the entire group. In contrast, the sample weight values depend on the personal data distribution:

5 $${\text{weight} }_{i}=\left\{\begin{array}{c}{\left(\frac{{A}_{i}{+N}_{i}}{{A}_{i}}\right)}^{0.8} if\, the\, slice\, has\, apnea \\ { \left(\frac{{A}_{i}{+N}_{i}}{{N}_{i}}\right)}^{0.8} if\, the\, slice\, has\, no\, apnea\end{array}\right.$$

where the ${A}_{i}$ and ${N}_{i}$ represent the number of the apnea and normal segments of the ith patient.

After EfficientNet was trained as an apnea classifier, we took the model without a fully connected layer as an OSA feature extractor in the per-record classification training task. Here, the parameters of the extractor did not need to be tuned again. The 1280 features, which are extracted from the patients’ whole night record through the preprocessing and extractor, would be used to train the machine learning model and enable it to screen the patient’s OSA level.

### Performance evaluation

In the following section, we employ four common metrics: accuracy, sensitivity, specificity, and area under the receiver operating characteristic curve (AUC) to evaluate the model’s performance. The definitions of the first three parameters are presented as Eqs. (6) to (8):

6 $$\text{Accuracy }\left(\text{ACC}\right)=\frac{TP+TN}{TP+TN+FN+FP},$$

7 $$\text{Sensitivity }\left(\text{SE}\right)=\frac{TP}{TP+FN},$$

8 $$\text{Specificity }\left(\text{SP}\right)=\frac{TN}{TN+FP},$$

where TP and TN represent true positive and true negative, respectively. FP and FN are false positive and false negative. The receiver operating characteristic curve (ROC) measures how accurately the model can categorize the data points as positive and negative. AUC measures the area underneath the ROC curve, which makes it easy to show how well the classifier will perform the given task [45].

### Supplementary Information

Supplementary material 1.

## Data Availability

The sleep data from CMUH sleep center cannot be shared publicly to protect the privacy of the subjects. However, upon request and subject to appropriate approvals, it will be shared by the corresponding author.

## Abbreviations

OSA: Obstructive sleep apnea
PSG: Polysomnography
AHI: Apnea–hypopnea index
ECG: Electrocardiography
SPO2: Peripheral oxygen saturation
EEG: Electroencephalography
EOG: Electrooculography
DL: Deep learning
CNN: Convolutional neural network
LSTM: Long short-term memory
CMUH: China Medical University Hospital
STFT: Short-time Fourier transform
ML: Machine Learning
BMI: Body mass indexes
AI: Apnea index
HI: Hypopnea index
TST: Total sleep time
GAP: Global average pooling
SVM: Support-vector machine
XGBoost: EXtreme Gradient Boosting
AUC: Area under the receiver operating characteristic curve
ACC: Accuracy
SE: Sensitivity
SP: Specificity
TP: True positive
TN: True negative
FP: False positive
FN: False negative
RRI: R–R interval
RA: R-peak amplitude
QA: Q-peak amplitude
RRID: RR interval first-order difference
ANN: Artificial neural network
