# Supplementary material for: EfficientNet-based machine learning architecture for sleep apnea identification in clinical single-lead ECG signal data sets
Source: Biomed Eng Online. 2024 Jun 20;23:57. doi: 10.1186/s12938-024-01252-w (PMC11188209; doi:10.1186/s12938-024-01252-w)
Supplement: Supplementary file 1 — Supplementary material 1. [file 12938_2024_1252_MOESM1_ESM.docx]

**Appendices**

| **Table A.1** | | | | | | | | |
| --- | --- | --- | --- | --- | --- | --- | --- | --- |
| Per-segment classification with different training and processing setting (data: CMUH) | | | | | | | | |
| DL model | Overlapped-slicing | Weight | Smoothing | Pretrained | ACC | SE | SP | AUC |
| EfficientNet | X | Class | - | 🗸 | 0.874 | 0.612 | 0.963 | 0.900 |
|  | X | Sample | - | 🗸 | 0.854 | 0.727 | 0.897 | 0.900 |
|  | 🗸 | Sample | X | X | 0.793 | 0.827 | 0.782 | 0.890 |
|  | 🗸 | Sample | X | 🗸 | 0.830 | 0.785 | 0.844 | 0.898 |
|  | 🗸 | Sample | 🗸 | X | 0.818 | 0.840 | 0.811 | 0.911 |
|  | 🗸 | Sample | 🗸 | 🗸 | 0.855 | 0.787 | 0.878 | 0.917 |

**Table A.2**

Results of sample weight and overlapping-slicing training in EfficinetNet B7

| Groups | Number of segments | ACC | SE | SP | AUC |
| --- | --- | --- | --- | --- | --- |
| ALL | (N=226613) | 0.855 | 0.787 | 0.878 | 0.917 |
| AHI < 5 | (N=90513) | 0.882 | 0.521 | 0.894 | 0.843 |
| AHI 5-15 | (N=60646) | 0.821 | 0.570 | 0.871 | 0.828 |
| AHI 15-30 | (N=30502) | 0.825 | 0.760 | 0.861 | 0.896 |
| AHI >30 | (N=44952) | 0.872 | 0.888 | 0.829 | 0.936 |
